# Synthesis of Non-Cytotoxic Poly(Ester-Amine) Dendrimers as Potential Solubility Enhancers for Drugs: Methotrexate as a Case Study

**DOI:** 10.3390/molecules15118082

**Published:** 2010-11-09

**Authors:** Delia Soto-Castro, Jorge A. Cruz-Morales, María Teresa Ramírez Apan, Patricia Guadarrama

**Affiliations:** 1 Instituto de Investigaciones en Materiales, Universidad Nacional Autónoma de México, Apartado Postal 70-360, CU, Coyoacán, Mexico, DF, 04510, Mexico; E-Mails: sotodelia@hotmail.com (D.S.C.); sirarmando@hotmail.com (J.A.C.M.); 2 Instituto de Química, Universidad Nacional Autónoma de México, Apartado Postal 70-213, CU, Coyoacán, Mexico, DF, 04510, Mexico; E-Mail: mtrapan@servidor.unam.mx (M.T.R.A.)

**Keywords:** non-cytotoxic dendrimers, solubility enhancer, methotrexate

## Abstract

This study describes the synthesis of two new families of dendrimers based on the esterification of *N*-alkylated 3-amine-1-propanol with two different cores, adipic acid (1^st^ and 2^nd^ generations) and ethylenediamine (generation 1.5), both with carboxylic acid end groups, offering a wide variety of further modifications at the periphery. According to the cytotoxic evaluation of the dendrimers and their possible degradation products within cell lines, these materials could be considered as innocuous. In preliminary studies, the synthesized dendrimers proved to be potential enhancers of solubility of highly hydrophobic drugs, like methotrexate, widely used in chemotherapy.

## 1. Introduction

After at least three decades of study, dendrimers remain as one of the most exciting and promising molecular architectures for a great variety of applications in important fields such as catalysis [1], alternative sources of energy [2,3], and biomedical applications for both medical diagnostics and therapeutic applications, among others [4,5,6,7,8]. In the area of drug delivery, there is a constant search of new vehicles for many drugs with serious solubility and cytotoxicity problems that make it difficult for them to reach their main targets. Recently, progress has been made in the new field of nanomedicine, where nanoscale engineered materials, such as PAMAM (polyamidoamine) dendrimers, are used in several medical applications, as well as dendritic scaffolds in biology [9,10].

**Figure 1:**
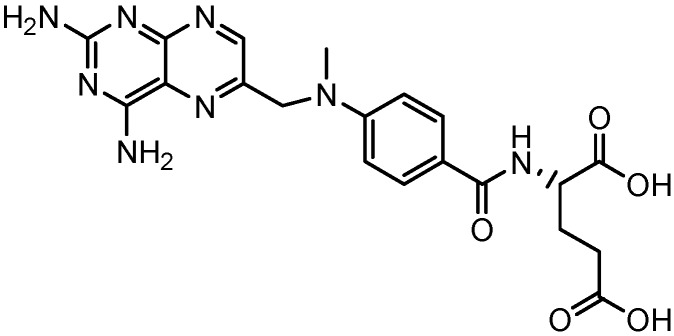
Chemical structure of methotrexate (MTX).

In this regard, the unique architecture and functionality of dendrimers at this scale make them exceptional carrier molecule candidates for use in medical applications. Thus, the present study describes the synthesis of two new families of poly(ester-amine) dendrimers, designed to be considered as flexible non-cytotoxic nanocarriers of poorly water-soluble drugs, like methotrexate (MTX, Figure 1), widely used in chemotherapy [11].

To evaluate the cytotoxicity of the dendrimers and their possible degradation products, assays on different cell lines were performed. In addition, these new materials were also tested as enhancers of MTX solubility in water. Preliminary theoretical simulations were carried out to visualize the hydrophobic/hydrophilic balance once MTX has interacted with the dendrimers.

## 2. Results and Discussion

Taking into account the key features that materials with bio-applications should have, we designed and synthesized dendritic compounds addressing three important elements: i) high flexibility to enforce the encapsulation events and to improve solubility; ii) biodegradability by the presence of ester groups, since they are cleaved in the body and, at the same time, induce formation of hydrogen bonds; and iii) non-cytotoxicity by selecting carboxylic acids as end groups (pKa ~5), which should be practically deprotonated at the blood’s pH (7.4), leading to the formation of anionic entities that prevent adhesion to the walls of blood vessels by electrostatic repulsions [12]. Dendrimers with anionic components have shown to be less toxic and less hemolytic than their cationic counterparts [13,14,15,16]. To avoid acidic solutions, the carboxylic end groups can undergo an acid-base reaction with NaOH to produce the corresponding carboxylates.

**Scheme 1:**
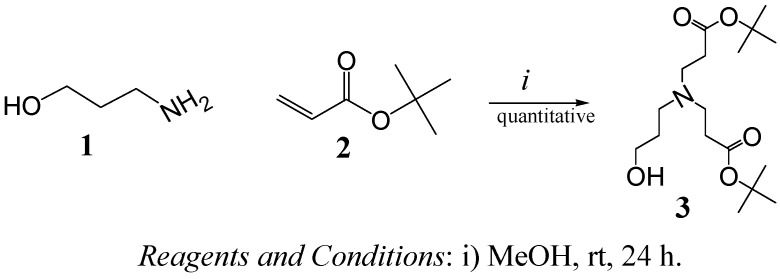
Synthesis of dendron **3**.

**Scheme 2:**
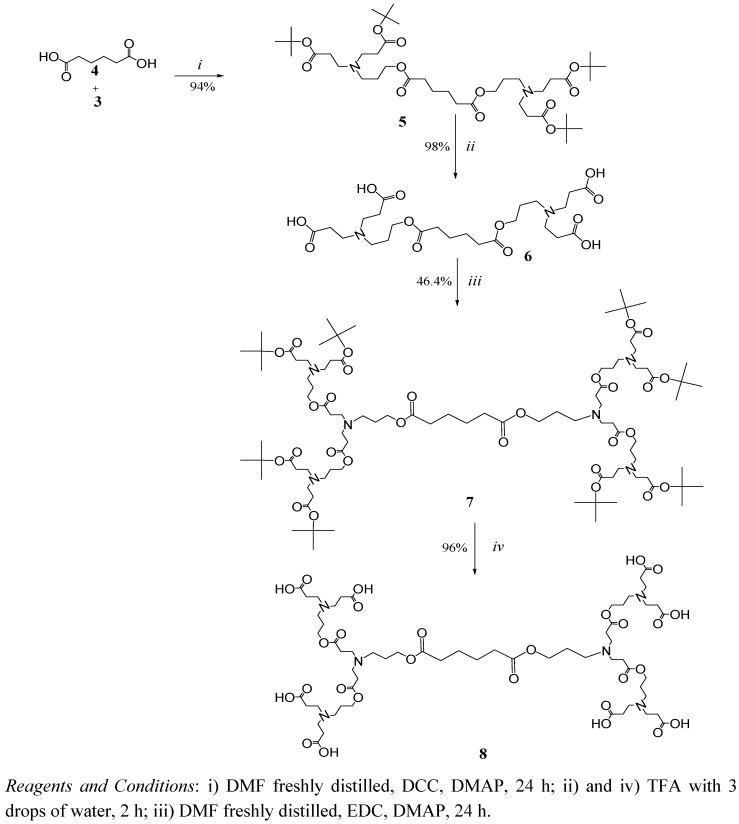
Synthesis of dendrimers with an adipic acid core

**Scheme 3:**
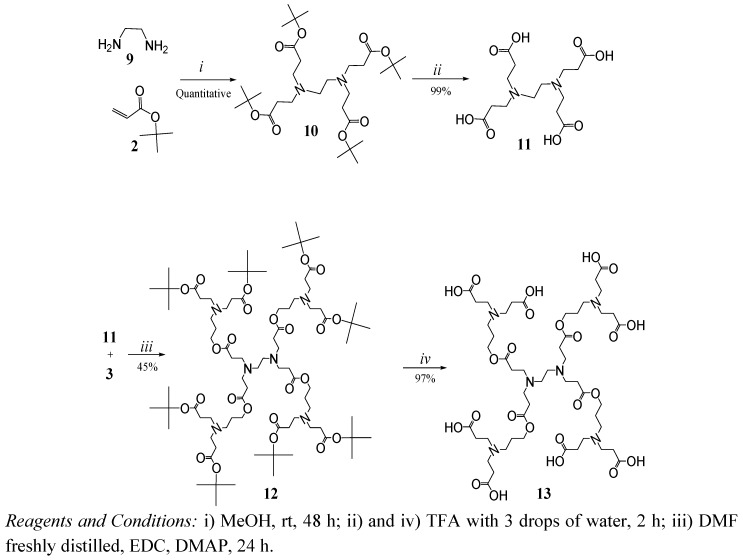
Synthesis of dendrimers with ethylenediamine core.

The synthesis of compound **3** [17] (Scheme 1), from 3-aminopropan-1-ol (**1**) and *tert*-butyl acrylate (**2**) enabled us to obtain a 1^st^ generation dendron bearing a single reactive point, a OH group, ready to be coupled via an esterification reaction to -COOH end groups of proposed cores (see Scheme 2 and Scheme 3). The reaction was allowed to proceed for 24 h, then the solvent and excess reactants were evaporated to give the product, quantitatively. The structure was corroborated by common spectroscopic techniques.

In a convergent fashion, dendron **3** was coupled to two different cores with terminal carboxylic acids, one is adipic acid **4** (Scheme 2) and the other is compound **11** (Scheme 3) obtained from the alkylation of ethylenediamine and their subsequent hydrolysis.

From Scheme 2, adipic acid and dendron **3** (1:6 mole ratio) were coupled in the presence of DCC/DMAP (dicyclohexylcarbodiimide/dimethylaminopyridine) to obtain compound **5** in high yield (96%) as an amber oil. The DMF solvent must be completely dried and freshly distilled; otherwise the yield drops to 30–35%. This new compound was characterized by ^1^H NMR, where the ratio of signals corresponding to the *tert-*butyl groups [36 H, 1.42 ppm (s)] was compared with those of the adipic core (8H) distributed in two groups of signals in 1.64 and 1.76 ppm. In the ^13^C-NMR spectrum, only those carbons related to ester groups in 171 and 173 ppm, corresponding to *tert*-butyl, and internal esters were observed, respectively. Finally, through FAB+ a *m/z* value of 773, corresponding to compound **5**, was obtained.

The hydrolysis of compound **5** with trifluoroacetic acid (TFA) takes place in a selective manner to generate compound **6** (G1 dendrimer with carboxylic acid terminals) in 98% yield. The structure was corroborated by ^1^H-NMR, where the signal in 1.42 ppm, associated with the *tert*-butyl groups, had disappeared.

The DCC/DMAP coupling system was used to couple dendron **3** to compound **6** in order to obtain compound **7** (Scheme 2), like the coupling of adipic acid to dendron **3**. Even though the DCC/DMAP coupling system usually gives excellent yields (86%, in this case), a serious inconvenience arose due to the presence of dicyclohexylurea (DCU), formed as by-product of the reaction with DCC, as the former was strongly encapsulated by the dendrimer (compound **7**), which was confirmed by ^1^H-NMR (signals in 3.21 ppm, related to DCU).

Failed attempts to revert the encapsulation were made by filtration, centrifugation, and dialysis with flexible and rigid membranes; therefore, a change in the coupling system was required. EDC (*N*-(3-dimethylaminopropyl)-*N*′-ethylcarbodiimide) was chosen instead of DCC, since its urea is much more polar and more easily removed than the DCU by column chromatography; although the yield dropped to 46.4%, as shown in Scheme 2. In spite of the latter inconvenience, the strong encapsulation of highly insoluble DCU provides preliminary evidence of the ability of these compounds to enhance the solubility of hydrophobic compounds.

Thus, compound **7** was also obtained with the EDC/DMAP coupling system after chromatographic purification with ethyl acetate/NH_4_OH (1% v/v) as eluent. The structure was confirmed by ^1^H-NMR, where the ratio of signals in 1.44 and 1.65 ppm corresponds to the new relation of *tert*-butyl protons and the more protected methylene protons present in the structure of the dendron. Through FAB+, a *m/z* of 1,801 was obtained.

Compound **7** was hydrolyzed in TFA to obtain dendrimer **8** with 96% yield. This compound is highly hygroscopic and completely soluble in water, a desirable characteristic in materials with potential application as drug carriers.

On account of their chemical structure, these compounds exhibit two characteristic FT-IR bands. One, broad and weak around 3,500-2,200 cm^-1^ attributed to COOH end groups and another band around 1,650 cm^-1^ attributed to the carbonyl of the –COO^-^···^+^NHR_3_ salt. The formation of this kind of salts can produce duplicity in the NMR signals; therefore, the addition of aqueous HCl (drops) can be required. The hydrolyzed compound **3** shows no more duplicity of signals, hence all NMR spectra of hydrolyzed compounds were recorded after addition of a few drops of HCl (see Supplementary Materials).

A second family of dendrimers was synthesized starting from the alkylation of ethylenediamine with *tert*-butyl acrylate (Scheme 3) to obtain compound **10** as a white solid in quantitative yield. The structure of this compound was verified by ^1^H-NMR with signals at 1.44 ppm (*tert*-butyl groups) and a singlet at 2.53 ppm corresponding to the symmetric ethylenediamine core.

As before, the carboxylic acid end groups were obtained after the hydrolysis of compound 10 with TFA to obtain compound **11** with 99% yield as a poorly water-soluble solid. Compound **11** is less flexible and has less polar groups in comparison with compound **6**, which exhibits the same number of terminal groups.

The coupling of compound **11** and dendron **3** allowed us to obtain compound **12**. Its ^1^H-NMR reveals a singlet at 1.43 ppm corresponding to *tert-*butyl groups and a *m/z* of 1601 was found by FAB+. Compound **12** was hydrolyzed to obtain dendrimer **13** (G1.5) in 97% yield as a highly hygroscopic foamy solid. The FT-IR bands at 3,600 to 2,200 cm^-1^ and the disappearance of the singlet at 1.43 ppm in the NMR confirmed the hydrolysis.

## 3. Biological Section

Since non-cytotoxicity is a necessary feature for any biomaterial, the harmlessness of dendrimers **8** and **13** and their possible degradation products (hydrolyzed compound **3**, and compounds 6 and 11) was evaluated by assays on human lymphocytic cells (MT2) [18]. The results of the assays (performed in triplicate), as well as the standard error, are shown in Table 1, where a sample of polyglycidol (synthesized in our lab, see Experimental) was included as reference parameter of innocuity [20]. Although the main interest for the biological study was the verification of non-cytotoxycity of the new materials in normal cell lines, additional assays on human cancer cell lines were carried out for forthcoming studies.

**Table 1:** Activity (%) of human cancer cell lines and human lymphocytic MT2 cells.^**a**^

Compound	MT2	U251	PC-3	K-563	HCT-15	MCF7	SKLU-1
**3** (hydrolyzed)	+3.4±2.0	10.6±1.2	+6.6±5.0	6.5±1.2	7.8±1.4	24.0±1.2	17.9±1.3
**4**	+3.2±1.5	+13.7±2.9	+3.7±0.8	+12.0±1.3	+6.5±1.0	+11.1±6.0	+5.1±0.5
**6**	+3.0±1.5	1.6±1.0	+5.2±0.2	15.3±5.8	+3.5±3.3	+21.4±5.4	+2.3±0.8
**8**	+6.1±1.0	9.8±0.4	12.6±3.6	7.7±0.0	+5.9±2.0	+10.2±2.2	2.4±0.4
**11**	6.7±2.2	19.7±5.0	+1.7±1.0	11.8±2.4	9.9±3.3	+9.8±4.2	+5.4±1.3
**13**	1.3±0.6	11.2±0.5	+9.6±2.1	1.3±0.3	+4.4±4.0	+6.6±1.2	2.1±0.1
Polyglycidol**^b^**	+5.8±0.6	1.6±0.1	+5.4±1.8	6.4±0.08	4.8±1.5	5.3±0.2	5.5±1.5

^a^: Cellular activity measured in presence of different compounds at 50 µM ± standard error. MT2: Human lymphocytes; Human cancer cell lines: U251 (human glyoblastoma); PC-3 (human prostatic adenocarcinoma); K562 (human chronic myelogenous leukemia cells); HCT-15 (human colorectal adenocarcinoma); MCF-7 (human mammary adenocarcinoma); SKLU-1 (human lung adenocarcinoma); **b**: MW = 4591; polydispersity = 1.8.

In Table 1 the + sign denotes cell growth compared with the control, which means that the compounds are non cytotoxic; and the values without + sign correspond to cell growth inhibition (%). From these results it can be observed that these new dendrimers, as well as their possible metabolites, exhibit cytotoxycity towards some cancer cell lines; however, they are harmless towards normal cells. The cytotoxycity observed for compound **11** and dendrimer **13** in normal cells is considered negligible.

Even though polyglycidol (reference compound) showed cytotoxycity in some cancer cell lines, it was also non cytotoxic towards normal cells (lymphocytes).

The performance of dendrimers **8** and **13** eventually might propitiate a synergistic effect [21] when the drug is included, since these materials already exhibit certain level of cytotoxycity toward some cancer cell lines. Concerning the possible metabolites **3** (hydrolyzed) and **11** (hydrosoluble molecules eliminable from the body), their differential effect in human glyoblastoma (U251) and human mammary adenocarcinoma (MCF-7) might be related to the selectivity of these cells, in terms of lipophilicity [22].

## 4. Dendrimers as Enhancers of Solubility: Preliminary Studies with MTX

MTX is an anticancer drug extensively used against breast and cervical cancer, although it is essentially insoluble in water at neutral pH [23]; therefore, it is an ideal candidate to be solubilized by dendrimers, ideally forming complexes. In order to avoid possible repulsive interactions owing to the dissociation of acid groups present in both, dendrimers and MTX, the dendrimer-drug complexes were formed in absence of water, initially grinding in a mortar the corresponding dendrimer with NaOH (equimolar amounts) to form the salt, followed by the addition of MTX to form the complex, as a paste, which was dissolved in deionized water up to 5 mL in a volumetric flask (see Experimental section). Water soluble complexes were obtained after sonication, centrifugation, and filtration of the precipitated MTX. The complexes remained stable at neutral pH during several days of storage in darkness.

Due to the very poor solubility of MTX in deionized water, our attempts to construct a calibration curve by UV-Vis at different concentrations failed, hence concentration values of MTX in the presence of dendrimers were unobtainable; however, from the measured absorbance values of dendrimer-MTX complex solutions in water in an appropriate dilution, and considering the Lambert-Beer law, it was possible to establish a ratio of concentrations to clearly show that both dendrimers, **8** and **13**, significantly improve MTX solubility in water. To determine such a ratio of concentrations, it was necessary first to measure at least one absorbance value corresponding to MTX in water, which was achieved by means of the formation of a saturated solution of MTX in deionized water, and making the required dilutions to apply the Lambert-Beer law.

After a dilution of a saturated solution (0.3 mL up to 5 mL) of MTX, an absorbance (*A_302_*) of 0.457 at 302 nm was obtained. Applying Equations 1 and 2 and by algebraic treatment yields the concentration of MTX in water as a function of the constant (*k*):

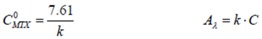
(1)

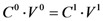
(2)


In a similar way, and making the appropriate dilutions (0.2 mL up to 5 mL), the measured absorbance values in water at 302 nm for the complexes formed between dendrimer **8** and **13** with MTX were 1.261 and 0.827 respectively.

Based on equations 1 and 2, and the maximum concentartion of MTX in water, the ratio of concentrations (the concentration of MTX in the presence of dendrimer (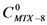) by the concentration of MTX in water alone (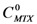)), can be expressed as follows:

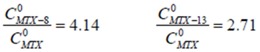



**Figure 2:**
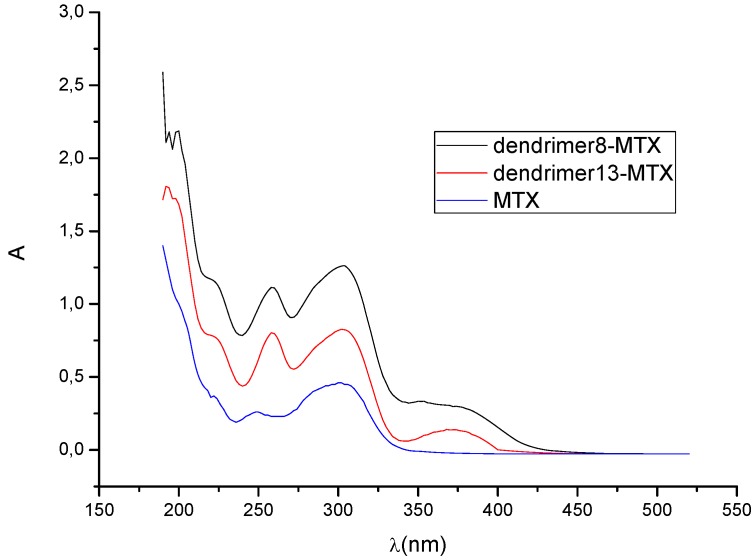
UV-VIS spectra of dendrimer8-MTX and dendrimer13-MTX complexes and MTX in deionized water. The relative concentrations with regard to MTX in water are 2.71 to **13**-MTX complex and 4.14 to **8**-MTX complex.

Thus, the presence of dendrimer **8** increases 4.14-times the concentration of MTX in water, while dendrimer **13** increases it 2.71-times. Figure 2 shows the UV-VIS spectra of MTX alone in water and dendrimer-MTX complexes. From Figure 2 a slight shift and best definition of maximum of MTX at 372 nm can be observed in the presence of dendrimers presumably due to dendrimer-MTX interactions via π-π ^*^ transitions.

With the experimental information described before it is possible to estimate a stoichiometry of interaction between dendrimers and MTX. Considering that the same initial concentration of each dendrimer (*C_D_*=1.7 mM) was used to form the complexes dendrimer-MTX, and comparing their performance to interact with MTX, we assumed a 1:1 stoichiometry for the dendrimer **8**-MTX complex since the largest increment of MTX concentration in water is achieved with dendrimer **8**.

Under this hypothesis, a 5:3 stoichiometry for dendrimer **13**-MTX complex was estimated, taking into account the obtained concentration ratios by algebraic manipulation (see Experimental). 

Considering the estimated stoichiometries, possible conformations of the dendrimer-MTX complexes were explored by conformational search calculations in aqueous medium (continuum dielectric constant = 81). The Monte Carlo multiple minimum method (MCMM) was used [24,25], where random changes were made in torsion angles during the search.

**Figure 3:**
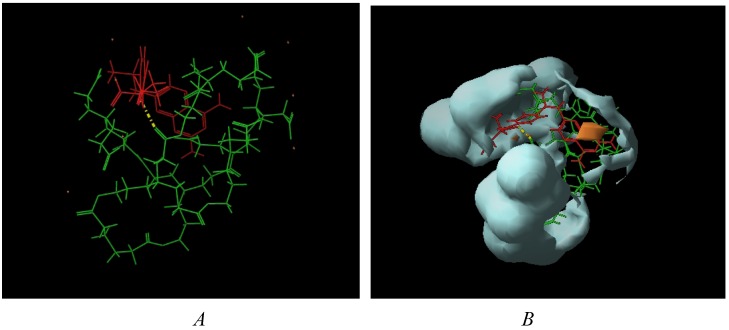
(a) dendrimer **8**-MTX; (b) Hydrophilic/hydrophobic balance.

Thus, considering, on one hand, carboxylate end groups (scenario at pH ~7), and, on the other hand, the experimentally observed complexation stoichiometry, ~1:1 and 5:3 complexes between dendrimer **8** and MTX and dendrimer **13** and MTX were constructed, respectively. From Figure 3a it can be suggested that the interaction dendrimer-drug is viable. Due to the flexible adipic core, the dendrimer exhibits a relaxed structure that surrounds the MTX and interacts with it via hydrogen bonds. In order to obtain additional information about the ability of dendrimers for isolating the hydrophobic MTX from polar environments (aqueous medium), surfaces that display hydrophobic and hydrophilic regions (in orange and blue respectively) were generated by an active-site mapping procedure that follows the Goodford algorithm [26].

In case of complex **8**-MTX (Figure 3b), the drug is visibly shielded by the dendrimer, with the anionic end groups exposed to the aqueous medium, forming a hydrophilic bag with the drug inside.

**Figure 4:**
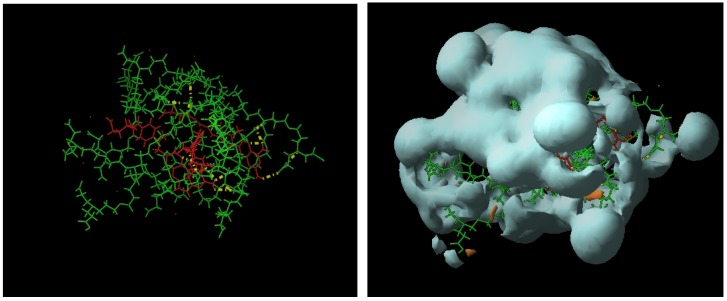
(a) dendrimer **13**-MTX (5:3); (b) Hydrophilic/hydrophobic balance.

In case of the less flexible dendrimer **13**, the proposed complexation stoichiometry of 5:3 was minimized to obtain the conformation shown in Figure 4a, where the group of dendrimeric molecules surrounds the MTX, and several hydrogen bonds are formed.

According to the hydrophobic/hydrophilic balance, a homogeneous protection of MTX in an isolated bag formed by the dendrimeric cluster is observed (figure 4b). Thus, even when the experimental observation is the most reliable evidence of the interaction between the dendrimers and MTX, these preliminary calculations offer an illustrative rationalization of the experimental results.

## 5. Conclusions

The synthesis of two new families of ester-amine dendrimers constructed by esterification between 3-amine-1-propanol, previously *N*-alkylated, and two different cores was carried out. All dendrimers are monodisperse, highly soluble in water and both dendrimers and their metabolites, are innocuous to human cells. Studies on solubilization of MTX place these materials as potential solubility enhancers of hydrophobic molecules. According to the dendrimer: MTX ratio, estimated by UV-VIS spectra, the stoichiometry of the complexes depends on the molecular flexibility, directly related to the flexibility of the cores. Finally, the preliminary calculations to explore the conformations of the complexes dendrimer-MTX showed an effective isolation of MTX, where the hydrophilic surface generated by the dendrimers can be related with the increase of solubility observed for the MTX in aqueous media.

## 6. Experimental

### 6.1. General

Unless stated otherwise, chemicals were obtained from commercial sources and used without further purification. DMF was dried over CaH_2_ (5% w/v) overnight, filtered and freshly distilled at reduced pressure prior to its use. Uncorrected melting points were determined on an electrothermal 9100 Fisher and NMR spectra were recorded on a Bruker Avance 400 instrument. FT-IR spectra were measured on a Nicolet 6700 spectrometer. MS (FAB) mass spectra were measured with a JEOL AX-505 spectrometer and by ESI with a Bruker Daltonic DataAnalysis 3.2. Molecules **8**, **13**, MTX, and the complexes were equilibrated by conformational search in aqueous media with a continuum dielectric constant of 81 (Force Field OPLS2001) [27]), using the Monte Carlo statistical method [28] included in the Macromodel software. The algorithm of multiple minima (MCMM) [24,25] without limits on the number of variable torsions allowed in the search was used. Hydrophobic and hydrophilic surfaces were calculated using Maestro [29] with a box of 6 Å.

#### 6.1.1. General Procedure for the Synthesis of *tert*-Butyl Ester Terminated Dendrimer

To an ice-cold suspension of carboxylic acid compound, a mixture of dendron (compound **3**) and DMAP in dry DMF (in 12 mL), a solution of EDC (1-ethyl-3-[(3-dimethylamino)propyl]-carbodiimide) or DCC (dicyclohexilcarbodiimide) in DMF were added dropwise. The reaction mixture was stirred at 0°C for 1 h and then at room temperature, monitored by thin layer chromatography (TLC) until no more changes were visible (for these products 24 h were enough). The solvent was removed under reduced pressure to give an amber oil, which was purified by column chromatography on silica gel using ethyl acetate/ammonium hydroxide (1% v/v) as eluent.

*(tert-Butyl 3-[(2-tert-butoxycarbonyl-ethyl)-(3-hydroxy-propyl)-amino]-propionate)* (**3**). In a round-bottom flask purged with nitrogen and maintained in darkness, 3-aminopropan-1-ol (**1**, 5 g, 66.57 mmol) was diluted in methanol (30 mL) and then *tert*-butyl acrylate (**2**, 22.183 g, 173.08 mmol) was added. The mixture was stirred at room temperature for 24 h, the excess of *tert*-butyl acrylate and solvent were removed at vacuum to give a colorless oil in quantitative yield. FT-IR (cm^-1^): 3,413 (OH), 2,976, 2,939 (C-H aliph.), 1,723 (C=O); ^1^H-NMR (CDCl_3_) δ: 1.39 (s, 18H, -C(**CH_3_**)_3_), 1.64 (q, 2H, -C***CH_2_***C-), 2.35 (t, 4H, -C***CH_2_***COO-), 2.57 (t, 2H, -C(***CH_2_***)N-), 2.69 (t, 4H, -N***CH_2_***C-) ppm;^ 13^C-NMR δ: (CDCl_3_, 77), 27.9 (-C(***CH_3_****)*_3_), 28.2 (-C(***CH_2_****)_2_*C-), 33.1 (-C***CH_2_***COO-), 49.2 (-N***CH_2_***C-), 52.7 (-C(***CH_2_***)_2_N-), 62.8 (HO**CH_2_**C-), 80.4(-COO***C***(CH_3_)_3_) and 171.6(-C**COO**C-) ppm.

*(4-Cascade:adipic acid [2-1,8]:(1-azabutylidyne):tert-butylpropyl ester)* (**5**). To an ice-cold suspension of adipic acid (**4**, 1g, 6.84 mmol) compound **3** (13.6 g, 41.05 mmol) and DMAP (0.167 g, 1.37 mmol) dissolved in dry DMF (12 mL) and DCC (3.38 g, 16.41 mmol) in DMF (5 mL) were added in portions. When the reaction was finished the mixture was purified, as described above to give **5** (4.97 g, 94%) as an amber oil. FT-IR (cm^-1^): 2,973, 2,933 (C-H aliph.), 1,722 (C=O); ^1^H-NMR (acetone d_6_) δ: 1.42 (s, 36H, -C(***CH_3_****)*_3_), 1.64 (m, 4H, -C(***CH_2_)_2_***C-), 1.76 (q, 4H, -C***CH_2_***C-), 2.34 (t, 12H, -C***CH_2_***COO-), 2.50 (t, 4H, -C***CH_2_***N-), 2.70 (t, 8H, -N***CH_2_***C-), 4.08 (t, 4H, -COO***CH_2_***C-) ppm; ^13^C-NMR δ: (acetone d_6_), 25 (-C(***CH_2_)_2_***C-), 27 (-C***CH_2_***COO-), 28 (-C(***CH_3_****)*_3_), 34 (-(CH_2_)_3_***CH_2_***COO-), 34 (-C***CH_2_***COO-), 49.8 (-N***CH_2_***C-), 50 (-C***CH_2_***N-), 63 (-COO***CH_2_***C-), 79 (-COO***C***(CH_3_)_3_), 171 (-***COO***(CH_3_)_3_) and 173 (-C***COO***C-) ppm; MS *m/z* (FAB+) 773.

*(8-cascade:adipic acid [2-1,8]:(1-azabutylidyne):(6-oxo-5-oxa-1-azaoctylidyne):tert-butylpropyl ester)* (**7**). To an ice-cold suspension of **6** (2.4 g, 4.39 mmol) compound **3** (11.65 g, 35.16 mmol) and DMAP (0.726 g, 5.86 mmol) in dry DMF (12 mL), and EDC (8.18 g, 52.69 mmol) in DMF (5 mL) were added in portions. When the reaction was finished, the mixture was purified as described above to give **7** (3.67 g, 46.4%) as an amber oil. FT-IR (cm^-1^): 2,973, 2,933 (C-H aliph.), 1,722 (C=O); ^1^H-NMR (MeOD) δ: 1.44 (s, 72H, -C(***CH_3_****)*_3_), 1.65 (m, 4H, -C(***CH_2_)_2_***C-), 1.76 (q, 12H, -C***CH_2_***C-), 2.33 (t, 28H, -C***CH_2_***COO-), 2.50 (t, 12H-C***CH_2_***N-), 2.70 (t, 24H, -N***CH_2_***C-), 4.08 (t, 12H, -COO***CH_2_***C-). ^13^C (δ (ppm)): (MeOD): 24.3 (-C(***CH_2_)_2_***C-), 25.4 (-C***CH_2_***C-), 26.6 (-C***CH_2_***C-), 27.5 (-C(***CH_3_****)*_3_), 32.2 (-C***CH_2_***COO-), 33.4 (-(CH_2_)_3_***CH_2_***COO-), 33.7 (-C***CH_2_***COO-), 49.6 (-N***CH_2_***C-, C***CH_2_***N-), 62.2 (-COO***CH_2_***C-), 79.3 (-COO***C***(CH_3_)_3_), 171.3 (-***COO***(CH_3_)_3_) 172.4 (-C***COO***C-) ppm; MS: *m/z* (FAB+) 1,801 and 1,803 by ESI.

*(4-Cascade:ethylenediamine[4-N,N,N´,N´]: tert-butyl propyl ester)* (**10**). In a round-bottom flask purged with nitrogen and maintained in darkness, ethylenediamine (2 g, 33.27 mmol) was diluted in methanol (30 mL), and then *tert*-butyl acrylate (22.183 g, 173.08 mmol) was added. The mixture was stirred at room temperature for 24 h, the excess of *tert*-butyl acrylate and solvent were removed at vacuum to give a white solid in quantitative yield (19 g). p.f. 42 ºC. FT-IR (cm^-1^): 2,940, 2,818 (C-H aliph.); 1,715 (C=O); ^1^H-NMR (MeOD) δ: 1.47 (s, 36H, -C(CH_3_)_3_); 2.36 (t, 8H, -C*CH_2_*COO-); 2.53 (s, 4H, -N(*CH_2_*)_2_N-); 2.73 (t, 8H, -N*CH_2_*C-) ppm; ^13^C-NMR δ: (MeOD); 27 (-C(***CH_3_****)*_3_); 33 (‑C***CH_2_***COO-); 55 (-N(***CH_2_***)_2_N-); 80 (-COO***C***(CH_3_)_3_); 172 (-***COO***(CH_3_)_3_) ppm.

*(4-Cascade:ethylenediamine[4-N,N,N´,N´]:(6-oxo-5-oxa-1-azaoctylidyne):tert-butylpropyl ester)* (**12**). To an ice-cold suspension of **11** (1.5 g, 4.3 mmol), compound **3** (11.51 g, 34.72 mmol) and DMAP (0.726 g, 5.68 mmol) in dry DMF (12 mL) and EDC (8.02 g, 51.66 mmol) in DMF (5 mL) were added in portions. When the reaction was finished, the mixture was purified as described above, to give **12** (3.1 g, 45%) as an amber oil. FT-IR (cm^-1^): 2,975, 2,818 (C-H aliph.) 1,723 (C=O); ^1^H-NMR (MeOD) δ: 1.43 (s, 72H, -C(***CH_3_****)*_3_); 1.74 (q, 8H, -C***CH_2_***C-); 2.36 (t, 16H, -C***CH_2_***COO-); 2.48 (t, 8H, ‑C***CH_2_***COO-); 2.52 (t, 8H, -N***CH_2_***C-); 2.56 (s, 4H, -N(***CH_2_***)_2_N-); 2.72 (t, 16H, -N***CH_2_***C-); 2.80 (t, 8H, -C***CH_2_***N-); 4.10 (t, 8H, -COO***CH_2_***C-) ppm; ^13^C-NMR (MeOD) δ: 25 (-C***CH_2_***C-); 27 (-C(***CH_3_****)*_3_); 33 (-C***CH_2_***COO-); 35 (-C***CH_2_***COO-); 48 (-N***CH_2_***C-); 49 (- N***CH_2_***C-); 50 (-C***CH_2_***N-); 55 (-N(***CH_2_***)_2_N-); 62 (-COO***CH_2_***C-); 80 (-COO***C***(CH_3_)_3_); 172 (-***COO***(CH_3_)_3_); 173 (-C***COO***C-)ppm; MS *m/z* (FAB+) 1,601 and 1,603 by ESI.

#### 6.1.2. General Procedure for the Hydrolysis of *tert*-Butyl Esters

*tert*-Butyl ester-terminated dendrimer (1 g) was dissolved in trifluoroacetic acid (TFA, 2 mL) with 3 drops of water. The hydrolysis was monitored by TLC. The TFA was evaporated under reduced pressure to give a viscous brown light oil, which was dissolved in methanol (10 mL) and evaporated under reduced pressure; this step was repeated from 3 to 5 times until no more TFA was present. As an alternative, the viscous oil can be triturated several times in hexane-dichloromethane (hex-DCM, 50/50) until no more TFA is present. Finally, the product was dried under high vacuum.

*(4-Cascade:adipic acid [2-1,8]:(1-azabutylidyne):propionic acid)* (**6**). Compound **5** (2 g, 2.58 mmol) was dissolved in TFA (4 mL). Once the general procedure was applied, the product was dried under high vacuum to give **6** (1.4 g, 98%) as a white, foamy and very hygroscopic product. FT-IR (cm^-1^): 2,220 to 3,600 (COOH broad and weak), 2,953 (C-H aliph), 1,738 and 1,662 (C=O); ^1^H-NMR (MeOH+HCl) δ: 1.66 (m, 4H, -C(***CH_2_)_2_***C-), 2.20 (q, 4H, -C***CH_2_***C-), 2.42 (t, 4H, -(CH_2_)_3_***CH_2_***COO -), 2.88 (t, 8H, -C***CH_2_***COO-), 3.48 (t, 8H, -N***CH_2_***C-), 3.74 (t, 4H-C***CH_2_***N-), 4.21 (t, 4H, -COO***CH_2_***C-) ppm; ^13^C-NMR δ: (MeOD): 23.0 (-C(***CH_2_)_2_***C-), 28.0 (-C***CH_2_***C-), 28.4 (-C***CH_2_***COO-), 33.0 (-(CH_2_)_3_***CH_2_***COO -), 48.5 (-N***CH_2_***C-), 49.5 (-C***CH_2_***N-), 61 (-COO***CH_2_***C-), 172 (-C***COO***C-) and 171 (-C***COOH***) ppm; MS *m/z* (ESI) 550.9.

*(8-Cascade:adipic acid [2-1,8]:(1-azabutylidyne):(6-oxo-5-oxa-1-azaoctylidyne):propionic acid)* (**8**). Compound **7** (1.5 g, 0.83 mmol) was dissolved in TFA (3 mL). Once the general procedure was applied, the product was dried under high vacuum to give **8** (1.08 g, 96%) as a white, foamy, very hygroscopic product. FT-IR (cm^-1^): 2,220 to 3,600 (COOH broad and weak), 2,943 (C-H aliph), 1,720 and 1,662 (C=O); ^1^H-NMR (MeOD) δ: 1.65 (q, 4H, -C(***CH_2_)_2_***C-), 1.96 (q, 12H, -C***CH_2_***C-), 2.25 (t, 4H, -OC**C*H_2_***(CH_2_)***_2_CH_2_***CO-), 2.86-2.96 (m, 24H, -NCH_2_**CH_2_**COO-), 3.28 (m, 4H,-C***CH_2_***N-), 3.41-3.47 (2t, 24H, -N***CH_2_***C-), 3.64 (t, 8, -C***CH_2_***N_f_-), 4.20 (t, 12H, -COO***CH_2_***C-)ppm; ^13^C-NMR δ: 25.2 (‑C(***CH_2_)_2_***C-), 26.7 (-OC**C**H_2_(CH_2_)***_2_C***H_2_CO-), 29.2, 34.4(-C***CH_2_***COO-), 50.8 (-N***CH_2_***C-,-C***CH_2_***N-), 60.6 (-COO***CH_2_***C-), 172.5, 175.7 (-C***COO***C-) and 172.5 (-***COO***H) ppm; MS *m/z* (ESI) 1354.

*(4-Cascade:ethylenediamine[4-N,N,N´,N´]: propionic acid)* (**11**). Compound **10** (1 g, 1.89 mmol) was dissolved in TFA (2 mL). Once the general procedure was applied, the product obtained was triturated in dichlorometane-hexane (50/50) several times until compound **11** (0.66 g, 99%), a white solid, was precipitated. FT-IR (cm^-1^): 2,256 to 3,120 (COOH broad and weak); 2,992-3,011 (C-H aliph), 1,709, 1,651 (C=O); ^1^H-NMR (D_2_O) δ: 2.85 (t, 8H, -C***CH_2_***COOH), 3.46 (t, 8H, -N***CH_2_***C-), 3.72 (s, 4H, -N(***CH_2_***)_2_N-) ppm; ^13^C-NMR (D_2_O) δ: 28.3(-C***CH_2_***COO-), 47.8(-N(***CH_2_***)_2_N-), 50.3 (-N***CH_2_***C-) and 174.0 (-C**COOH**) ppm.

*(4-Cascade:ethylenediamine[4-N,N,N´,N´]:(6-oxo-5-oxa-1-azaoctylidyne):propionic acid)* (**13**). Compound **12** (1 g, 0.62 mmol) was dissolved in TFA (2 mL). Once the general procedure was applied, the product was dried under high vacuum to give compound **13** (0.70 g, 97%) as a slightly brown, foamy, very hygroscopic product. FT-IR (cm^-1^): 2,220 to 3,600 (COOH broad and weak), 2,981 (C-H aliph), 1,724, 1,660 (C=O); ^1^H-NMR (MeOD + HCl) δ: 2.92 (q, 8H, -C***CH_2_***C-), 2.88 (t, 16H, -C***CH_2_***COO-), 2.69 (t, 8H, -C***CH_2_***COO-), 3.28 (s, 4H, -N(***CH_2_***)_2_N-), 3.43 (t, 8H, -N***CH_2_***C-), 3.64 (t, 16H, -N***CH_2_***C-), 3.84 (t, 8H, -C***CH_2_***N-), 4.21 (t, 8H, -COO***CH_2_***C-) ppm; ^13^C-NMR δ: (MeOD+HCl): 26.9 (-C***CH_2_***C-), 29.6 (-C***CH_2_***COO-), 50.7 (-C***CH_2_***N-, (-N***CH_2_***C- ), 54.2 (-N(***CH_2_***)_2_N-), 60.5 (-COO***CH_2_***C-), 171.9 (-C***COO***C-) and 172.5 (-***COOH***) ppm; MS *m/z* (ESI) 1154.

#### 6.1.3. Polyglycidol (Reference Compound for Biological Assays).

In a round bottom flask under N_2 _atmosphere, a solution of potassium methoxide (CH_3_OK) was prepared with potassium (0.8 g, 11.3 mmol) in previously distilled methanol (15 mL), and the solution was heated at 60 °C during 10 min. Afterwards, a solution of glycidol monomer (9.5 mL, 0.1427 mol) in methanol (5 mL) was added dropwise during 3 h. The mixture was heated at 85 °C for 24 h. Drops of 10% HCl were added to the cold solution until a pH = 7 was reached; next, the solution was filtered and evaporated under vacuum to give a yellow pale oil product. M_w_ = 4591, PDI = 1.8, FT-IR (cm^-1^) 3,261 (OH), 2,877 (CH_2_,CH), 1,059 and 1,127 (C-O); ^1^H-NMR (D_2_O) δ: 3.08-3.68 (m, CH, CH_2_), 2.8–2.85 (m, CH,CH_2_ oxirane ring); ^13^C-NMR (D_2_O) δ: 58.7-62.9 (CH_2_OH), 79.4-78.1 (C-HOR), 71.7–73.7(CH_2_OR) and 71.0-68.8 (CHOH) ppm.

### 6.2. Cell Lines Culture and Culture Medium

The dendrimers were screened *in vitro* (in triplicate) against human cancer cell lines: HCT-15 (human colorectal adenocarcinoma), MCF-7 (human mammary adenocarcinoma), K562 (human chronic myelogenous leukemia), U251 (human glyoblastoma), PC-3 (human prostatic adenocarcinoma), SKLU-1 (human lung adenocarcinoma), cell lines were supplied by the National Cancer Institute (USA). Besides human lymphocytes MT2 cell lines, human tumor cytotoxicity was also determined by using the protein-binding dye sulforhodamine B (SRB) in microculture assays to measure cell growth, as described in the protocols established by the NCI [30]. The cell lines were cultured in RPMI-1640 medium supplemented with 10% fetal bovine serum, 2 mM L-glutamine, 10,000 units/mL penicillin G sodium, 10,000 μg/mL streptomycin sulfate, 25 μg/mL amphotericin B (Gibco), and 1% non-essential amino acids (Gibco). They were maintained at 37 °C in a humidified atmosphere with 5% CO_2_. The viability of the cells used in the experiments exceeded 95%, as determined with trypan blue. 

### 6.3. Cytotoxicity Assay

Cytotoxicity after treatment of the cancer cell lines and normal cells with the test compounds was determined using the protein-binding dye sulforhodamine B (SRB) in microculture assays to measure cell growth, as described in Monks *et al.*, 1991). The cells were removed from the tissue culture flasks by treatment with trypsin, and diluted with fresh media. From these cell suspensions, 100 μL, containing 5,000—10,000 cells per well, were pipetted into 96-well microtiter plates (Costar) and the material was incubated at 37 °C for 24 h in a 5% CO_2 _atmosphere. Subsequently, a solution of the dendrimers obtained by diluting the stocks (100 μL) was added to each well. The cultures were exposed for 48 h to the compound at a concentration of 50 µM. After the incubation period, cells were fixed to the plastic substratum by the addition of 50 μL of cold 50% aqueous trichloroacetic acid. The plates were incubated at 4 °C for 1 h, washed with tap H_2_O, and air-dried. The trichloroacetic-acid-fixed cells were stained by the addition of 0.4% SRB. Free SRB solution was then removed by washing with 1% aqueous acetic-acid. The plates were then air-dried, and the bound dye was solubilized by adding 10 mM unbuffered Tris base (100 μL). The plates were placed on a shaker for 5 min, and the absorption was determined at 515 nm using an ELISA plates reader (Bio-Tex Instruments).

### 6.4. Formation of Dendrimer-MTX Complexes

A general procedure for complex formation involved, firstly, the salt formation of the corresponding dendrimer with NaOH (equimolar amounts), grinding in an agate mortar without solvent, followed by the addition of five equivalents of MTX to obtain a dendrimer-salt/MTX paste. This paste was dissolved in deionized water until reaching a 5 mL volume in a volumetric flask to give a water-soluble complex. The complex solution was sonicated for 20 min. and allowed to equilibrate in darkness overnight. Afterwards, the solution was centrifuged in a Cole Parmer 17250 centrifuge for 10 min. at 3400 rpm. The supernatant was filtered through an ANOTOP 25 syringe filter with a 400 nm pore size. 

To determine the solubility of MTX in water, 5 mg of MTX was placed in a 5 mL volumetric flask, the solution was stirred for 30 min., sonicated for 20 min. and allowed to equilibrate in darkness overnight. Then, the solution was centrifuged for 10 min. at 3400 rpm. The supernatant was filtered through an ANOTOP 25 syringe filter with a 400 nm pore size. A dilution of 0.3 mL up to 5 mL was made to obtain a reading using the Lambert-Beer law. All data were recorded in triplicate in a UV-VIS spectrometer. The absorbance of MTX in deionized water was 0.457.

#### 6.4.1. Dendrimer 8-MTX Complex

Compound **8** (11.7 mg, 0.866 × 10^-5^ mol) was ground with NaOH (2.7 mg, 6.93 × 10^-5^ mol) in a mortar, then MTX (25.5 mg, 5.61 × 10^-5^ mol) was added and ground for 20 min. This paste was dissolved in deionized water in a volumetric flask up to 5 mL. The solution was sonicated, centrifuged, and filtered according to the general procedure. In order to get a UV-VIS spectrum, a dilution of 0.2 mL up to 5 mL was made and the absorbances at 302 was recorded in triplicate. The absorbance was 1.261.

#### 6.4.2. Dendrimer 13-MTX Complex

Compound **13** (14 mg, 0.866 × 10^-5^ mol) was ground with NaOH (2.7 mg, 6.93 × 10^-5^ mol) in an agate mortar; immediately MTX (25.5 mg, 5.61 × 10^-5^ mol) was added and ground for 20 min. This paste was dissolved in deionized water in a volumetric flask up to 5 mL. The solution was sonicated, centrifuged, and filtered according to the general procedure. In order to get a UV-VIS spectrum, a dilution of 0.2 mL up to 5 mL was made and the absorbances at 302 was recorded in triplicate. The absorbance was 0.827.

#### 6.4.3. Calculation of Complex Stoichiometry

Starting from 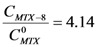; isolating *C_MTX-8_* and dividing by *C_D_*, we have: 
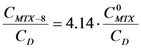
. If 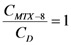 and *C_D_* = 1.7 mM, 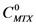 will be: 
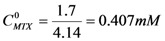
. With this result and evoking the concentration ratio for dendrimer 13, we have:

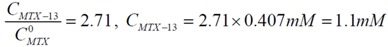



Thus, an estimated stoichiometry of 5:3 for dendrimer13-MTX complex can be obtained, multiplying and dividing by 3 to get a integer numbers, the follow expression:

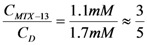


## Supplementary Materials

Supplementary materials can be seen at http://www.mdpi.com/1420-3049/15/11/8082/s1.
