# Supplementary material for: Synthesis of Non-Cytotoxic Poly(Ester-Amine) Dendrimers as Potential Solubility Enhancers for Drugs: Methotrexate as a Case Study
Source: Molecules. 2010 Nov 9;15(11):8082–97. doi: 10.3390/molecules15118082 (PMC6259256; doi:10.3390/molecules15118082)
Supplement: Supplementary file 1 [file molecules-15-08082-s001.pdf]

Molecules **2010**, *15*, 8082-8097; doi:10.3390/molecules15118082

Synthesis of Non-Cytotoxic Poly(Ester-Amine) Dendrimers as Potential Solubility Enhancers for Drugs: Methotrexate as a Case Study

Supplementary Materials

From ^{13}C spectrum of compound **3** (hydrolyzed with trifluoracetic acid), we can see the signals corresponding to protonated and non-protonated species: When drops of HCl are added, the duplicity disappeared.

^{13}C spectrum without HCl

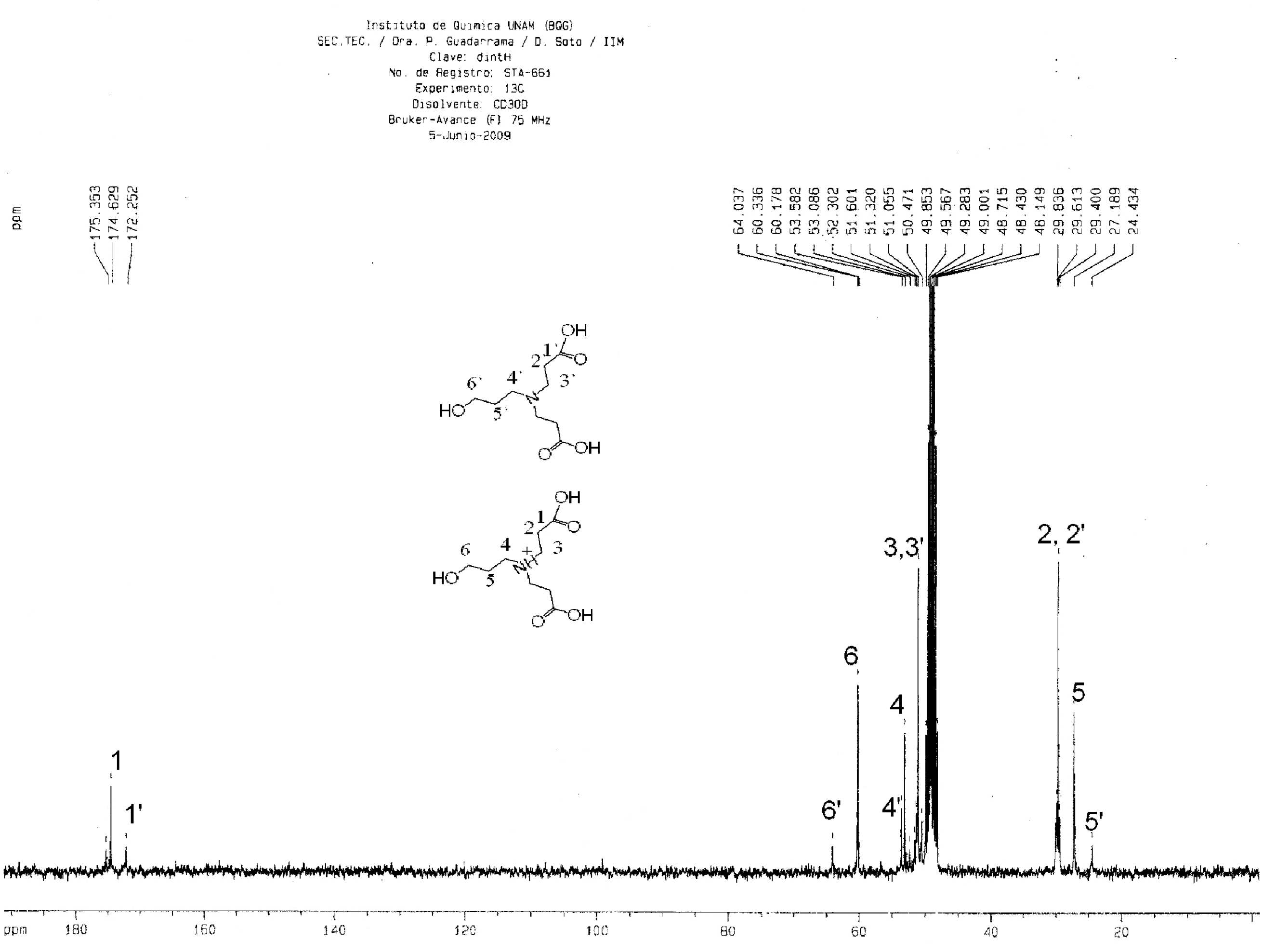

^{13}C spectrum with HCl

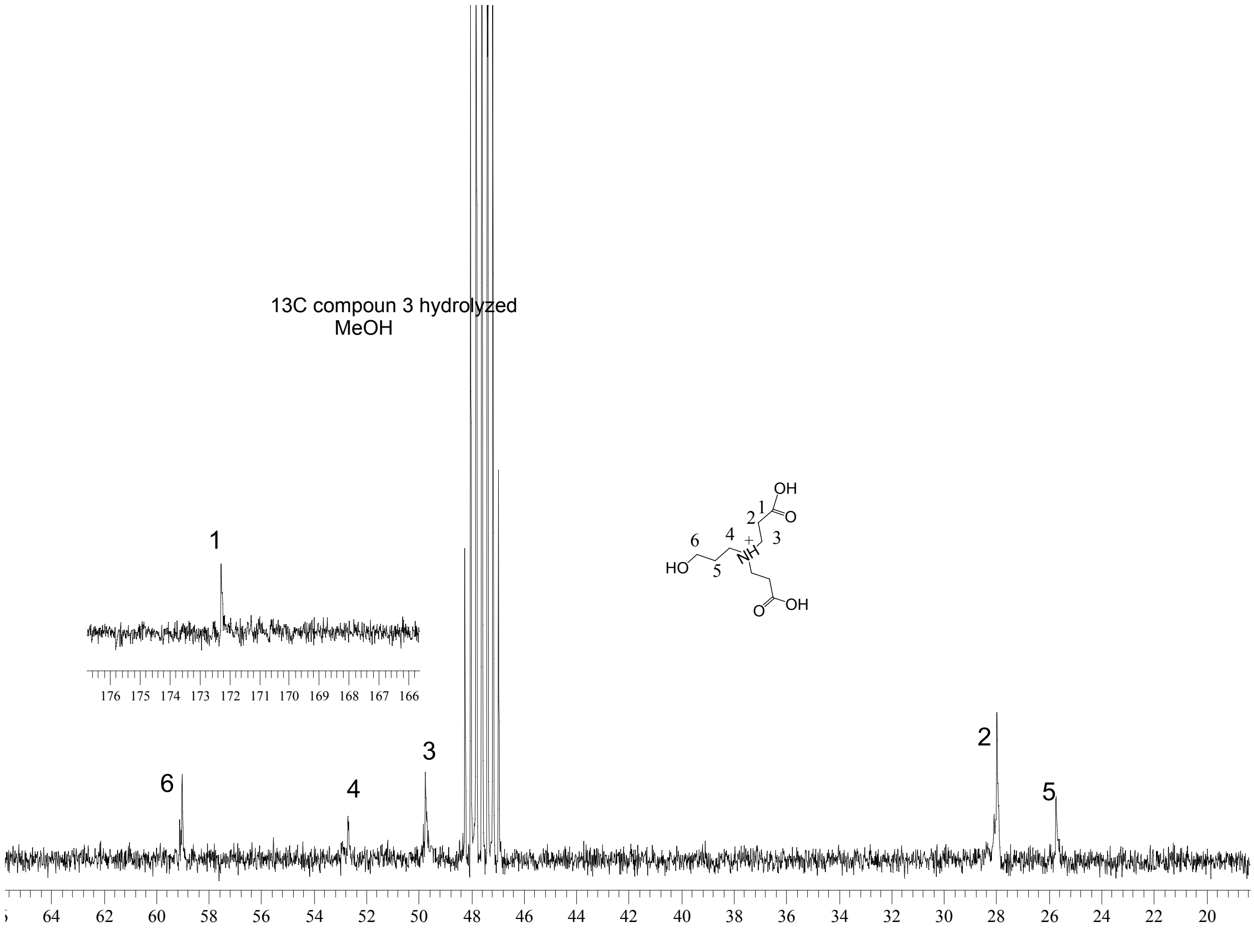

^{1}H RMN spectra of some synthesized compounds

Compound 5

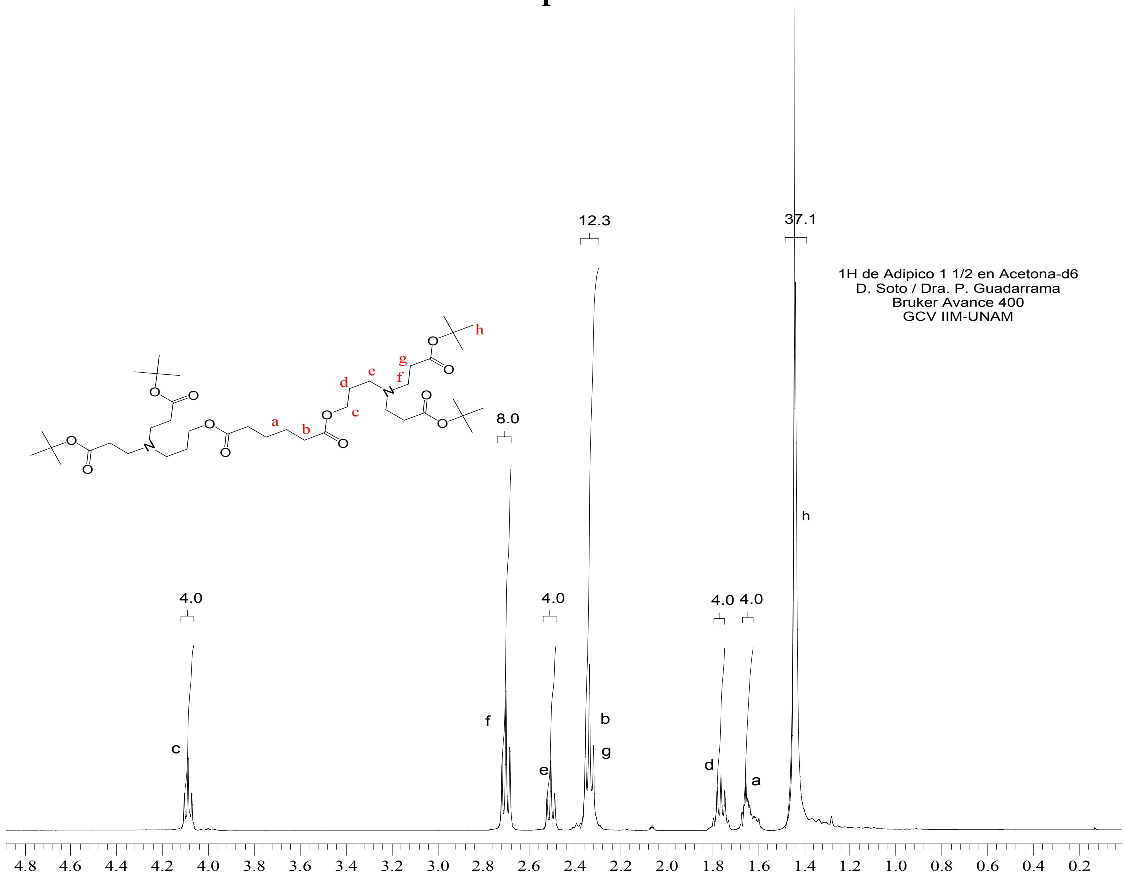

Compound 6

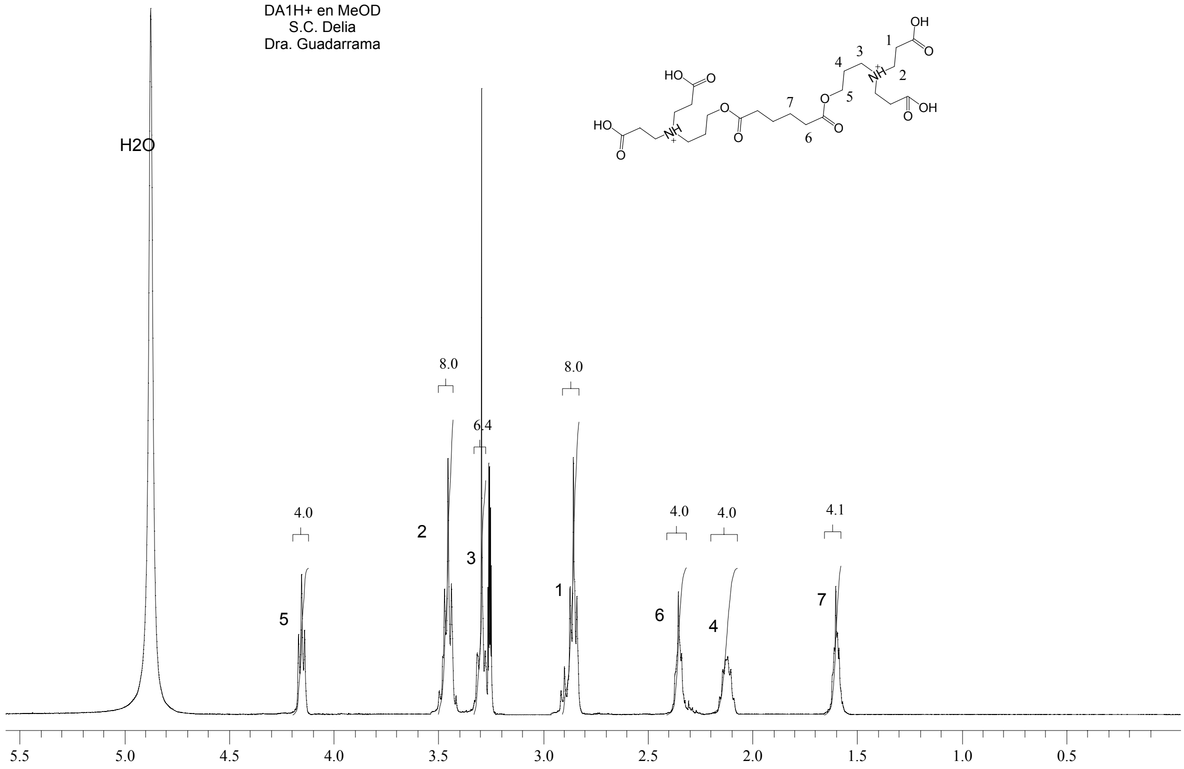

Compound 7

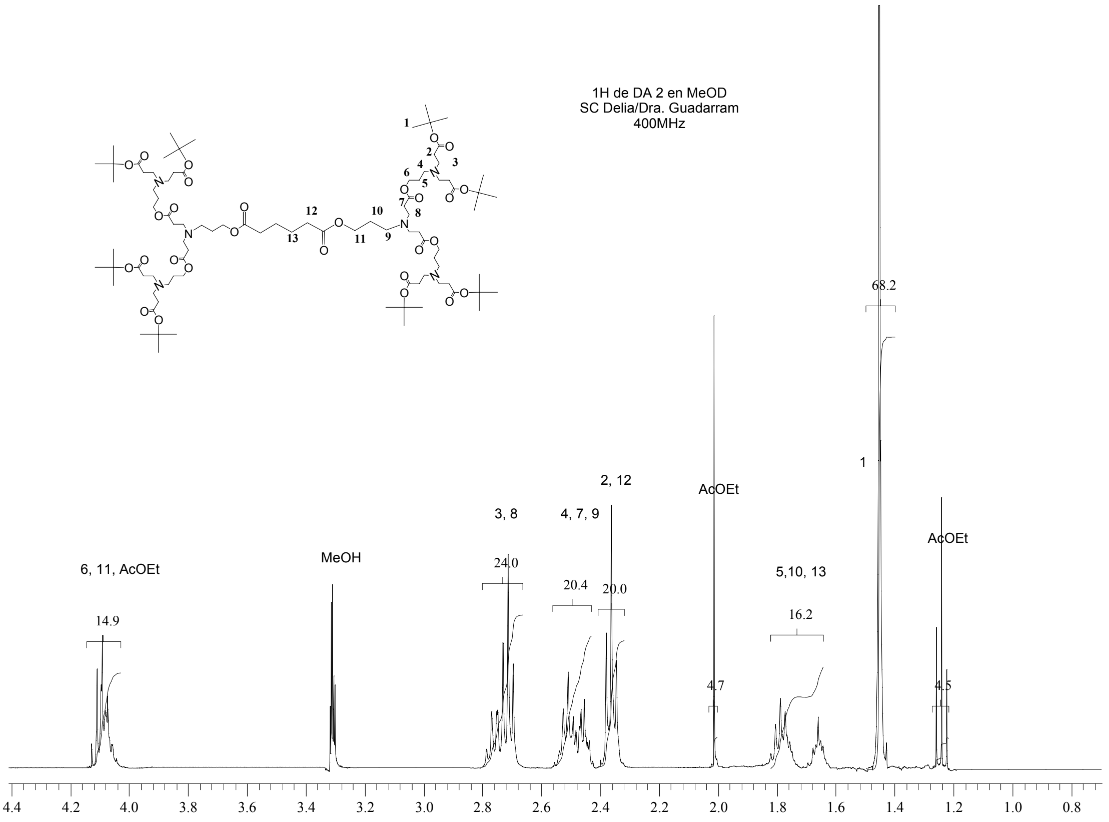

Compound 8

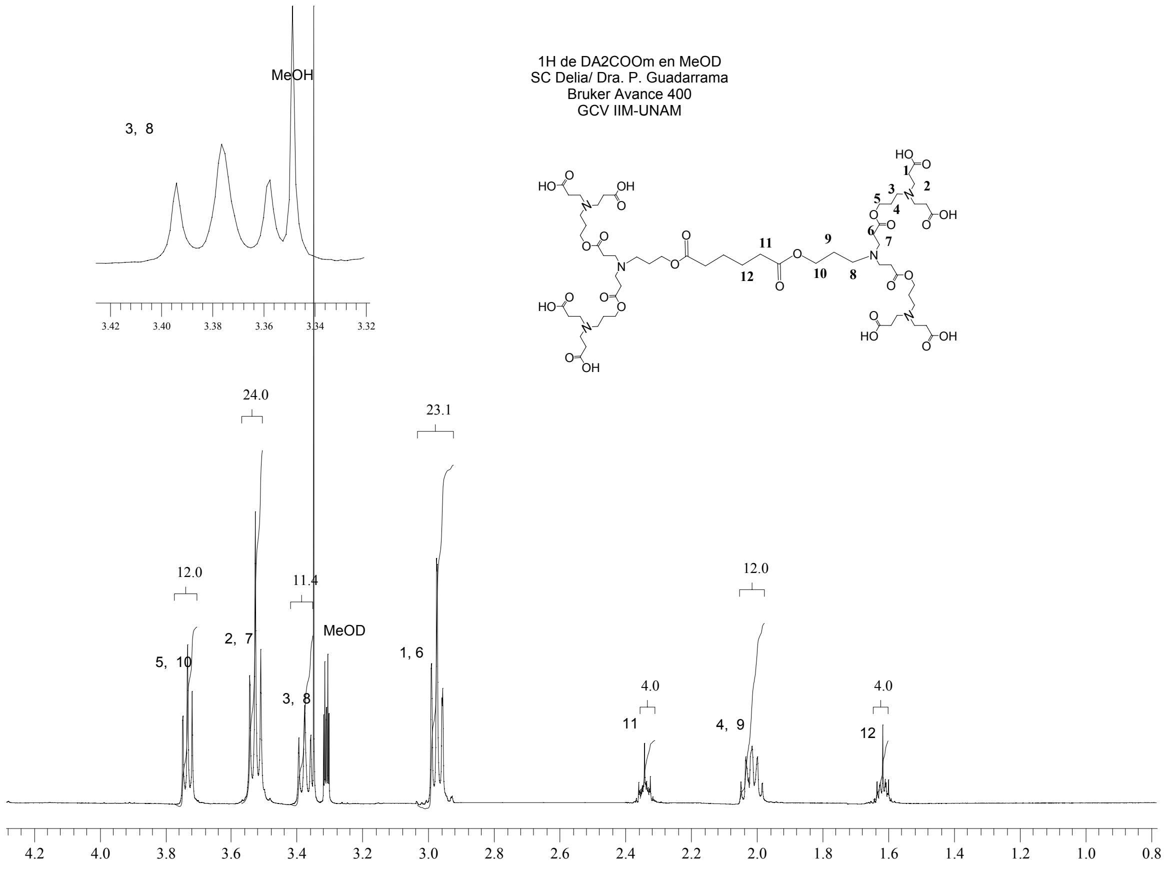

Compound 11

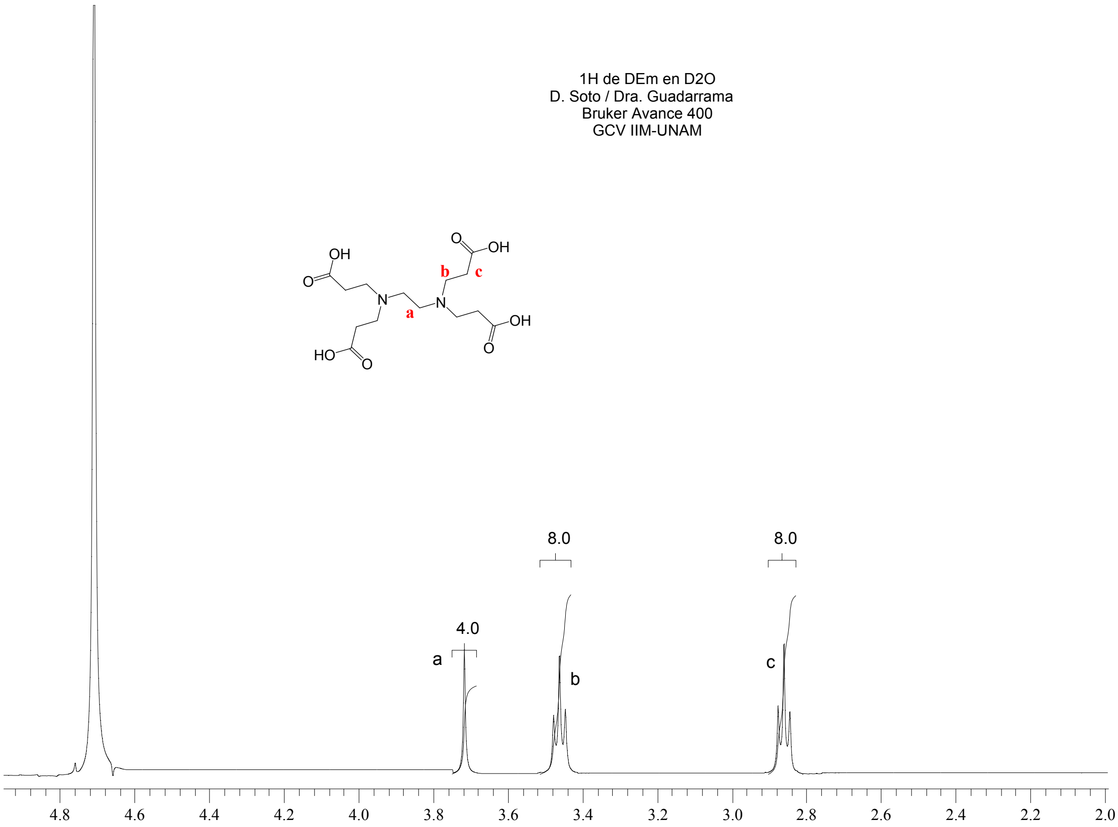

Compound 12

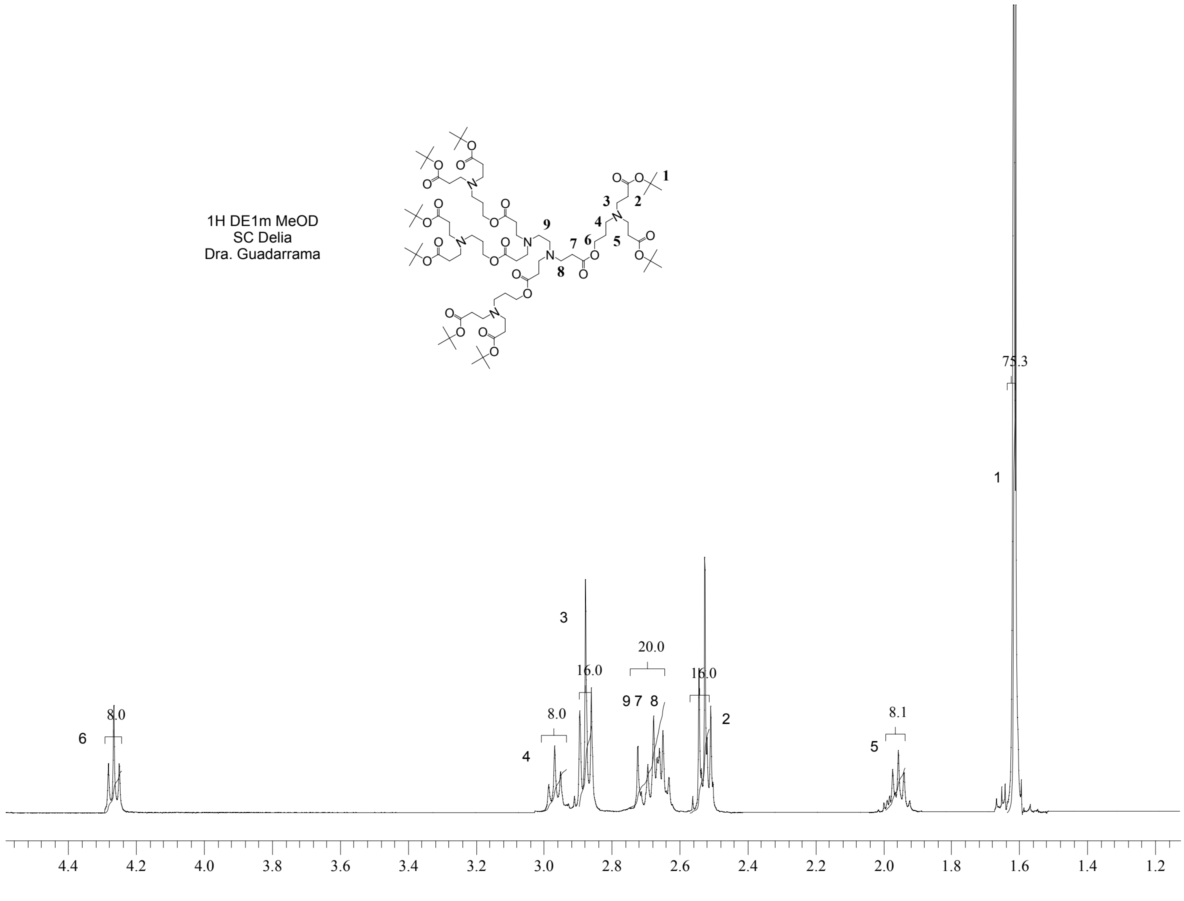

Compound 13

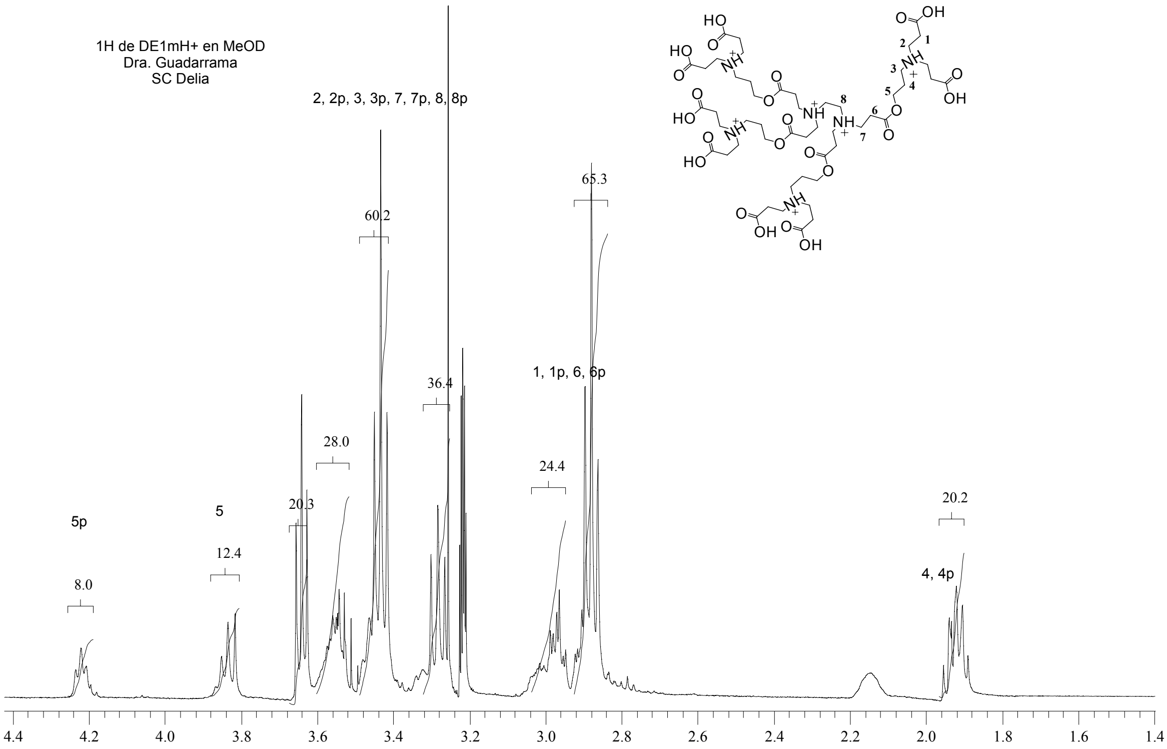
1H de DE1mH+ en MeOD
Dra. Guadarrama
SC Delia
2, 2p, 3, 3p, 7, 7p, 8, 8p
1, 1p, 6, 6p
5p
5
4, 4p
8.0
12.4
20.3
28.0
60.2
36.4
24.4
65.3
20.2
4.4
4.2
4.0
3.8
3.6
3.4
3.2
3.0
2.8
2.6
2.4
2.2
2.0
1.8
1.6
1.4
